# Facile Synthesis of Silver Nanowires with Different Aspect Ratios and Used as High-Performance Flexible Transparent Electrodes

**DOI:** 10.1186/s11671-017-2259-6

**Published:** 2017-08-07

**Authors:** Qingwen Xue, Weijing Yao, Jun Liu, Qingyong Tian, Li Liu, Mengxiao Li, Qiang Lu, Rui Peng, Wei Wu

**Affiliations:** 1 Laboratory of Printable Functional Nanomaterials and Printed Electronics, School of Printing and Packaging, Wuhan University, Wuhan, 430072 People’s Republic of China; 2 Shenzhen Research Institute of Wuhan University, Shenzhen, 518057 People’s Republic of China

**Keywords:** Ag NWs, Tailorable aspect ratios, Flexible transparent electrodes, Low-temperature welding

## Abstract

Silver nanowires (Ag NWs) are the promising materials to fabricate flexible transparent electrodes, aiming to replace indium tin oxide (ITO) in the next generation of flexible electronics. Herein, a feasible polyvinylpyrrolidone (PVP)-mediated polyol synthesis of Ag NWs with different aspect ratios is demonstrated and high-quality Ag NWs transparent electrodes (NTEs) are fabricated without high-temperature thermal sintering. When employing the mixture of PVP with different average molecular weight as the capping agent, the diameters of Ag NWs can be tailored and Ag NWs with different aspect ratios varying from ca. 30 to ca. 1000 are obtained. Using these as-synthesized Ag NWs, the uniform Ag NWs films are fabricated by repeated spin coating. When the aspect ratios exceed 500, the optoelectronic performance of Ag NWs films improve remarkably and match up to those of ITO films. Moreover, an optimal Ag NTEs with low sheet resistance of 11.4 Ω/sq and a high parallel transmittance of 91.6% at 550 nm are achieved when the aspect ratios reach almost 1000. In addition, the sheet resistance of Ag NWs films does not show great variation after 400 cycles of bending test, suggesting an excellent flexibility. The proposed approach to fabricate highly flexible and high-performance Ag NTEs would be useful to the development of flexible devices.

**Electronic supplementary material:**

The online version of this article (doi:10.1186/s11671-017-2259-6) contains supplementary material, which is available to authorized users.

## Background

Flexible transparent electrodes (FTEs) play an important role in the next generation of flexible electronics [1–4]. FTEs can be applied to many optoelectronic devices as conductive components, involving touch screens [5, 6], portable solar cells [7, 8], organic light-emitting diodes (OLEDs) [9–11], fuel cell electrode [12–17], sensors [18, 19], PM filter [20], transparent heaters [21, 22], and wearable electronics [23, 24]. The dominant transparent electrodes (TEs) used currently is indium tin oxide (ITO) owing to the low sheet resistance (<100 Ω/sq) and high transmittance (>80%). But its intrinsic brittleness limits the applications in flexible electronics. Moreover, it requires high temperature deposition process and is challenged by the scarcity of indium [25–27]. Therefore, several new conductive films with good flexibility and optical transparency, such as metal grids [2, 28, 29], carbon nanotubes (CNTs) [30–33], graphene [34–36], Ag NWs [5, 37–41], Cu NWs [42, 43], conductive polymers [44, 45], and hybrids of these [46–48], have been fabricated, striving to replace ITO. Among these candidates, Ag NWs films have been investigated extensively in both the scientific and industrial institutions, owing to the excellent electrical conductivity and high optical transparency. In addition, Ag NWs exhibit outstanding flexibility and stretchability, which is the one of the appealing advantage to fabricate stretchable transparent conductors than fragile ITO [49–51]. Moreover, the solution-processed Ag NWs films are more cost-effective than ITO. All of these properties make Ag NWs films become promising alternatives to ITO for the applications in flexible electronics.

However, several issues need to be addressed to commercialize Ag NWs films as FTEs. Firstly, Ag NWs with different aspect ratios need to be facilely synthesized in controlled manner because the alluring properties of Ag NWs films deeply rely on the dimensions of Ag NWs and a well-designed length and diameter are of very importance for different applications [52, 53]. Generally, polyol process is the most widely used method to prepare Ag NWs. Ran et al. [54] synthesized thin Ag NWs with aspect ratios larger than 1000 by using the mixed PVP with the average molecular weight of 58,000 and 1,300,000 as the capping agent. However, the influence of the aspect ratios on the optoelectronic performance of Ag NTEs was not carefully investigated in their work. Although Ding et al. [55] prepared Ag NWs with different diameters varying from 40 to 110 nm and fabricated Ag NTEs with a transmittance of 87% and a sheet resistance of ca.70 Ω/sq, many parameters need to be simultaneously adjusted to control the diameters of Ag NWs and the optoelectronic performance of the as-obtained Ag NTEs would not be satisfactory. Li et al. [56] synthesized thin Ag NWs with diameters of 20 nm through altering the concentration of bromide. And they have fabricated high-quality Ag NWs films with a transmittance of 99.1% at 130.0 Ω/sq. Ko et al. [57] developed a multistep growth method to synthesize very long Ag NWs over several hundred micrometers and the fabricated films demonstrated superior transmittance of 90% with sheet resistance of 19 Ω/sq. The optoelectronic performance of these Ag NWs films are comparable to or even better than those of ITO films. But the minimum aspect ratio of Ag NWs, which has the ability to fabricate TEs rivaling commercial ITO in terms of sheet resistance and transmittance, is still uncertain. Therefore, it is necessary to synthesize Ag NWs with various aspect ratios and study their influence on the optoelectronic performance of Ag NWs films.

Furthermore, the electronic conductivity of Ag NWs films is relatively poor, resulting from the high nanowire junction resistance [58]. In the polyol synthesis of Ag NWs, PVP, as the surfactant, adsorbs on the surface of Ag NWs, resulting in insulated contact between the wires in the random network [59, 60]. Consequently, different physical and chemical post-processes, involving thermal annealing [38, 39, 61, 62], mechanical press [63], nanosoldering with conductive polymers [64], plasmonic welding [65], laser nanowelding [66–68], and integration with other materials [60], have been explored to reduce the junction resistance. Among these post-treatments, thermal annealing at almost 200 °C is usually employed. It is incompatible with flexible plastic substrates which cannot withstand high temperature, and hence limits the applications of Ag NWs films in flexible optoelectronic devices.

Herein, a series of Ag NWs with different aspect ratios varying from ca. 30 to ca. 1000 are controllably synthesized and used to fabricate high conductive and transparent Ag NTEs. First, Ag NWs are prepared by facile PVP-mediated polyol process where the mixture of PVP with different average molecular weight can efficiently reduce the diameters. Subsequently, the as-synthesized Ag NWs with different aspect ratios are employed to fabricate Ag NWs films without high-temperature annealing, respectively. And the corresponding optoelectronic performance are comparative investigated. The best sheet resistance and parallel transmittance can achieve 11.4 Ω/sq and 91.6% when the aspect ratios reach almost 1000. Moreover, the sheet resistance of as-fabricated Ag NWs films is nearly constant after inner-bending and outer-bending tests.

## Methods

### Materials and Chemicals

Silver nitrate (AgNO_3_, AR) and anhydrous ethanol (C_2_H_5_OH, AR) were purchased from Sinopharm Chemical Reagent Co., Ltd. Copper (II) chloride dehydrate (CuCl_2_·2H_2_O, AR) and PVP (MW≈58,000, marked as PVP-58) were purchased from Shanghai Aladdin Reagents Co., Ltd. Ethylene glycol (EG, 98%) and PVP (MW≈10,000, 40,000 and 360,000, marked as PVP-10, PVP-40, and PVP-360, respectively) were purchased from Sigma-Aldrich. Deionized water (18.2 MΩ) was used in the whole experiments.

### Synthesis of Ag NWs

Ag NWs with different aspect ratios are prepared by a facile one-pot PVP-mediated polyol process. Typically, 0.170 g of AgNO_3_ is dissolved in 10 mL of EG under magnetic stirring. Then, 0.15 M of PVP-40 and 0.111 mM of CuCl_2_·2H_2_O mixed solution in 10 mL of EG is added dropwise to the above solution. Afterwards, the mixture is transferred into Teflon-lined stainless steel autoclave with a capacity of 50 mL and heated at 160 °C for 3 h. After cooling down to room temperature naturally, pure Ag NWs are obtained by centrifugation at a speed of 2500 rpm for 5 min and washed three times with ethanol and deionized water. Finally, the products are dispersed in ethanol for further characterization and application. Moreover, the concentration and average molecular weight of PVP are very important to control the morphology and size of products. Therefore, different types of PVP molecules are simultaneously used to regulate the diameters of Ag NWs in the polyol process. Detailed experimental parameters are listed in Additional file 1: Table S1, nominated as S1–S13, respectively.

### Fabrication of Ag NTEs

Polyethylene terephthalate (PET) with a thickness of 150 μm is cut to pieces with the dimension of 20 × 20 mm. Briefly, the as-prepared Ag NWs are dispersed in ethanol (6 mg/mL), and 50 μL of Ag NWs solution is spin coated at 2000 rpm for 30 s on PET substrate. Finally, the Ag NWs films are heated to 140 °C for 15 min without any additional post-process treatments. The aspect ratios of Ag NWs, rotation speed, concentration, and volume of Ag NWs solution are investigated to fabricate high-quality NTEs. Regarding to the repeated spin coating, each volume of Ag NWs solution is altered to 25 μL and the rotation speed is set to 2000 rpm. A time interval in each spin coating is needed to volatilize the ethanol. Other parameters are same as the aforementioned processes.

### Characterization and Performance Test

Scanning electron microscopy (SEM) images are recorded using a cold field-emission SEM (Hitachi S-4800). The transmission electron microscopy (TEM) and the high-resolution TEM (HRTEM) images are obtained by using a JEOL JEM-2100F. The UV-vis absorption spectra of Ag NWs and the optical transmittance spectra of Ag NWs films are carried out on a Shimadzu UV-3600 spectrophotometer. The sheet resistance is measured at room temperature by using 4-point probe resistance tester (FP-001).

## Results and Discussion

**Fig. 1:**
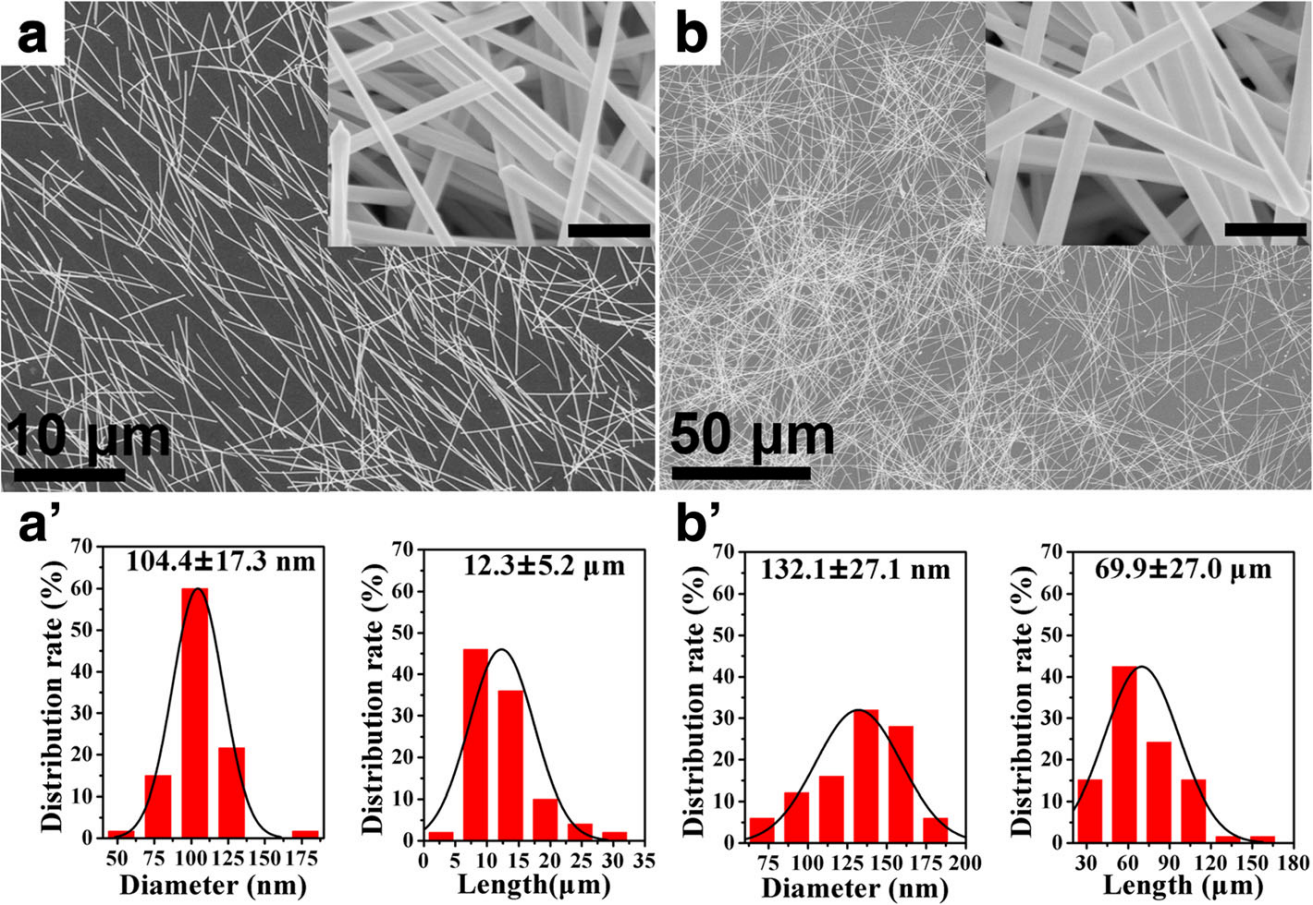
**a**, **b** SEM images of as-synthesized Ag NWs with PVP-40 and PVP-360, respectively. Both the concentration of PVP are 0.15 M. **a**′ **b**′ Corresponding statistical distribution of diameter and length. (The *insets* in **a** and **b** are the corresponding SEM images with high magnification and all the *scale bars* are 500 nm)

Generally, Ag NWs are synthesized by polyol process in which PVP is employed as capping agent to ensure the growth of one-dimensional Ag NWs [69, 70]. During the synthesis, many parameters such as reaction temperature, stirring speed, PVP concentration, PVP chain length, additive agents, and ratio of chemicals can affect the yield and morphology of synthesized Ag NWs. For example, an inappropriate reaction temperature less than 110 °C or higher than 180 °C allows more Ag atom to form Ag nanoparticles (NPs) rather than Ag NWs [70, 71]. The length of synthesized Ag NWs increase as slowing down the stirring speed [72, 73]. In this paper, we mainly investigate the concentration of PVP and their average molecular weight on the effect of morphology and size of Ag NWs. The corresponding morphology and size distribution of Ag NWs are demonstrated in Fig. 1 and Additional file 1: Figure S1. Firstly, the concentration of PVP is increased from 0.05 M (sample S1, Additional file 1: Figure S1a) to 0.15 M (sample S2, Fig. 1a). The corresponding morphology of products is changed from near-spherical Ag NPs to pure Ag NWs with an average diameter of 104.4 nm and length of 12.3 μm. The mixture of Ag NWs and Ag NPs are observed when the concentration of PVP is increased to 0.25 M (sample S3, Additional file 1: Figure S1b). By further increasing the concentration of PVP to 0.55 M (sample S4, Additional file 1: Figure S1c), a large number of Ag NPs with different shapes (including near-sphere and triangular plate) are formed. The results indicate that a lower or higher concentration of PVP are not beneficial to produce pure Ag NWs, further resulting in the absence of Ag NWs. The formation of Ag NPs in the products upon changing the concentration of PVP can be attributed to the failure of anisotropic growth over the entire surface of multiply twinned nanoparticles (MTPs) [69, 74].

In addition, the influence of PVP with different molecular weight on the morphology and size of Ag NWs is also discussed. Only Ag NPs and aggregated nanorods are produced when using PVP-10 (sample S5, Additional file 1: Figure S1d). When employing separately PVP-58 (sample S6, Additional file 1: Figure S1e) and PVP-360 (sample S7, Fig. 1b), the corresponding morphology and size of products are changed from stubby Ag NWs (with average diameter of 235 nm and length of 6.7 μm) to high aspect ratio Ag NWs (with average diameter of 132.1 nm and length of 69.9 μm). According to the abovementioned results from samples S2, S5, S6, and S7, the average molecular weight of PVP not only plays a vital role in the morphology formation of Ag NWs but also has a significant influence on the diameter and length of Ag NWs products. The influence of PVP with different average molecular weight on the morphology and size of Ag NWs can be ascribed to three factors: (i) PVP as the capping agent prefers to adsorb on the side faces of MTPs [69]. The strong chemical adsorption promotes the growth of long Ag NWs [75]. (ii) The steric effect of PVP capping layer allows silver atoms to deposit on the side faces through the gap between adjacent PVP molecules, further resulting in the formation of thick Ag NWs [54]. (iii) The high viscosity of PVP with high average molecular weight in EG solution would slow down the growth rate, which are benefit to form MTPs [76, 77]. As a result, the low average molecular weight of PVP, like as PVP-10, would not efficiently adsorb on the (100) crystal faces to restrict the lateral growth. Meanwhile, the small steric effect and low viscosity would not prevent the aggregation of silver nanostructures. PVP with high molecular weight, like as PVP-360, possesses strong chemical adsorption on the side faces to produce long Ag NWs. But the large steric effect of PVP-360 would lead to the increase of diameter.

**Fig. 2:**
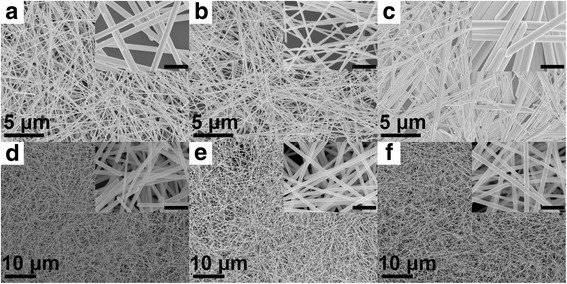
SEM images of Ag NWs synthesized using different mixed PVP molecules. **a** PVP-40:PVP-58 = 2:1, **b** PVP-40:PVP-58 = 1:1, **c** PVP-40:PVP-58 = 1:2, **d** PVP-40:PVP-360 = 2:1, **e** PVP-40:PVP-360 = 1:1, **f** PVP-40:PVP-360 = 1:2, respectively. All the total concentration of PVP are 0.15 M, and different PVP molecules are mixed at molar ratio. (The *insets* in **a**–**f** are the corresponding SEM images with high magnification, and all the *scale bars* are 500 nm)

In order to obtain high aspect ratios of Ag NWs, the adsorption strength and steric effect should be reached to a state of balance in the PVP-mediated system. Therefore, the mixed PVP molecules at different molar ratios are employed as capping agent and the corresponding morphology and size distribution of Ag NWs are showed in Fig. 2 and Additional file 1: Figure S2. When mixing PVP-58 with PVP-40 at the molar ratio of 1:1, Ag NWs with average diameter of 47.5 nm and length of 16.1 μm are obtained. While the molar ratio of PVP-40 and PVP-58 is adjusted to 1:2 or 2:1, the diameter of Ag NWs is increased. In addition, the aspect ratios of Ag NWs dramatically enlarge when mixing PVP-40 with PVP-360 because the diameters are reduced significantly. When the molar ratio of PVP-40 and PVP-360 is 1:1, the aspect ratios reach almost 1000 and the diameters have a more uniform distribution as shown in Fig. 2e.

**Scheme 1:**
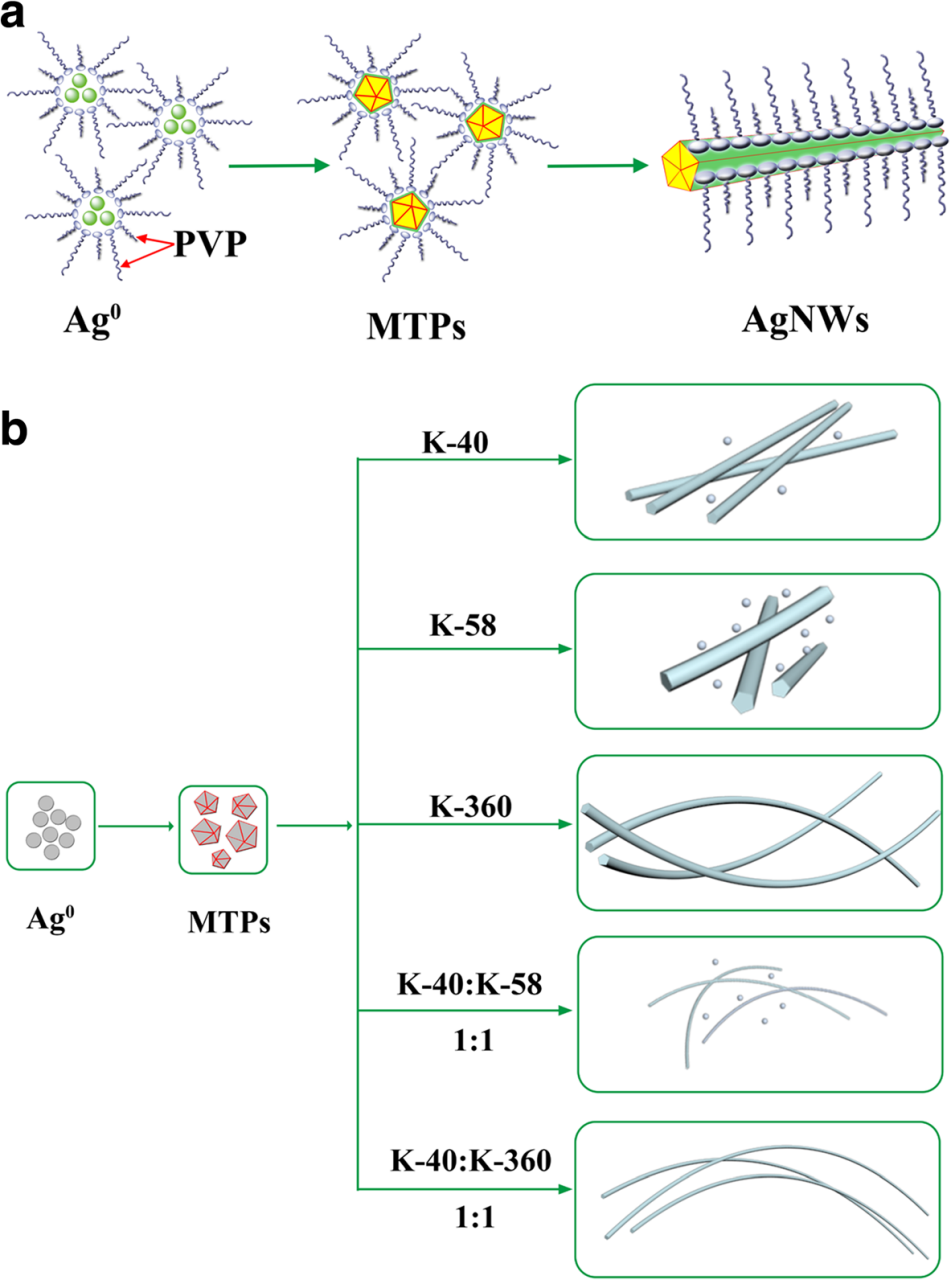
**a** Schematic illustration of the growth mechanism of Ag NWs using mixed PVP with different chain length. **b** Different aspect ratio Ag NWs are obtained by the PVP-mediated polyol process

The influence of mixed PVP with different chain length on the diameters of Ag NWs could be interpreted briefly in Scheme 1a. The long-chained PVP molecules can retard the lateral growth of Ag NWs owing to the strong adsorption to the (100) facets. The large steric effect, resulting from the long chains, brings a relatively large distance between adjacent PVP molecules. Ag atoms can still deposit on the surface of Ag NWs by diffusion through the gap between adjacent PVP molecules, and thick Ag NWs are produced. When using the mixed PVP with different chain length, the short-chained PVP can fill the gap between long-chained PVP. Therefore, the (100) facets can be passivated more efficiently, leading to the formation of smaller Ag seeds and thinner Ag NWs [76]. As shown in Scheme 1b, Ag NWs with typical aspect ratios are obtained in our work. It could be conjectured that higher aspect ratio Ag NWs may be produced through this experimental route.

**Fig. 3:**
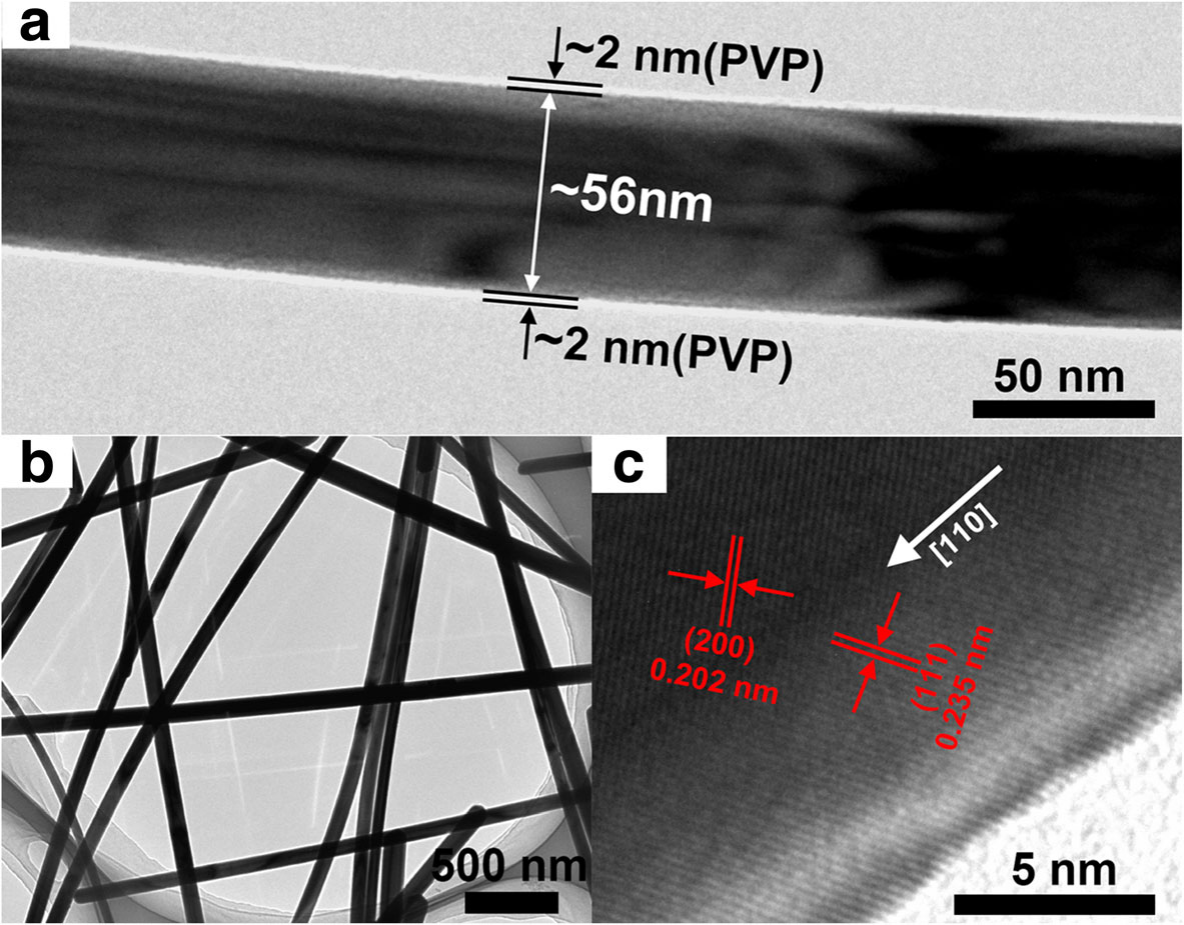
TEM (**a**, **b**) and HRTEM (**c**) images of Ag NWs synthesized by mixing PVP-40 with PVP-360 (at a molar ratio of 1:1)

The microstructure and morphology of Ag NWs are characterized by TEM and demonstrated in Fig. 3a, b. The single nanowire is coated by the thin PVP layer with a thickness of ca. 2 nm. Figure 3c shows the HRTEM image of Ag NWs with a good crystalline structure. The HRTEM image clearly exhibits that the spaces between periodic fringes are 0.235 and 0.202 nm, in good correspondence with the crystal plane spaces for (111) and (200) planes of face-centered cubic (fcc) Ag. Meanwhile, Ag NWs grow along the [110] direction, as marked by the white arrow, and it is similar to the results in the earlier reports [70, 76].

**Fig. 4:**
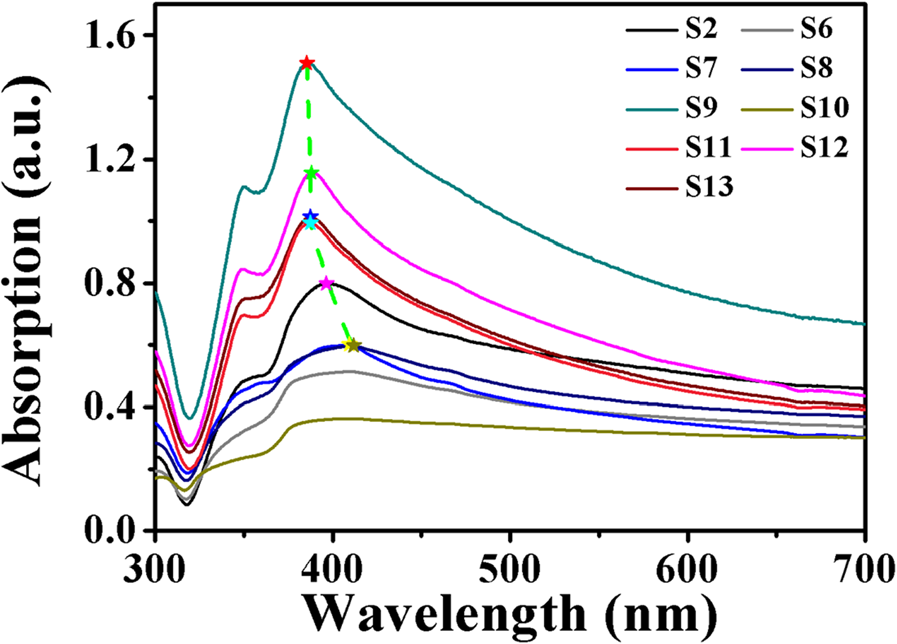
UV-visible absorption spectra of as-prepared Ag NWs with different diameters

As shown in Fig. 4, the UV-visible absorption spectra of as-prepared Ag NWs are different from that of the quasi-spherical Ag NPs. The spectra of Ag NWs appear double characteristic peaks. A shoulder peak located at around 350 nm could be ascribed to the plasmon resonance of bulk silver film [70, 78]. The second peak could be attributed to the transverse plasmon mode of Ag NWs, and the peak position is related to the dimensions of silver nanostructures [79]. While the peak at around 570 nm, resulting from the longitudinal plasmon resonance, is absent in the spectra because the aspect ratios of as-prepared Ag NWs are far more than 5 [70, 80]. In addition, as marked by the dashed green line, the second peak has a shift to red with the increase of diameters. However, it is noteworthy that there is no obvious peak when the diameters of Ag NWs become larger. For Ag NWs from sample S6 (average diameter of 235 nm) and S10 (average diameter of 222.8 nm), the absorption intensity maximums locate at the wavelength of 408.5 and 406.5 nm, respectively. They are smaller than the peak wavelength of Ag NWs with smaller diameters from sample S7 (average diameter of 132.1 nm, the peak wavelength is 412 nm), indicating the detachment of red-shifted tendency of the right peak wavelength with larger diameters.

**Fig. 5:**
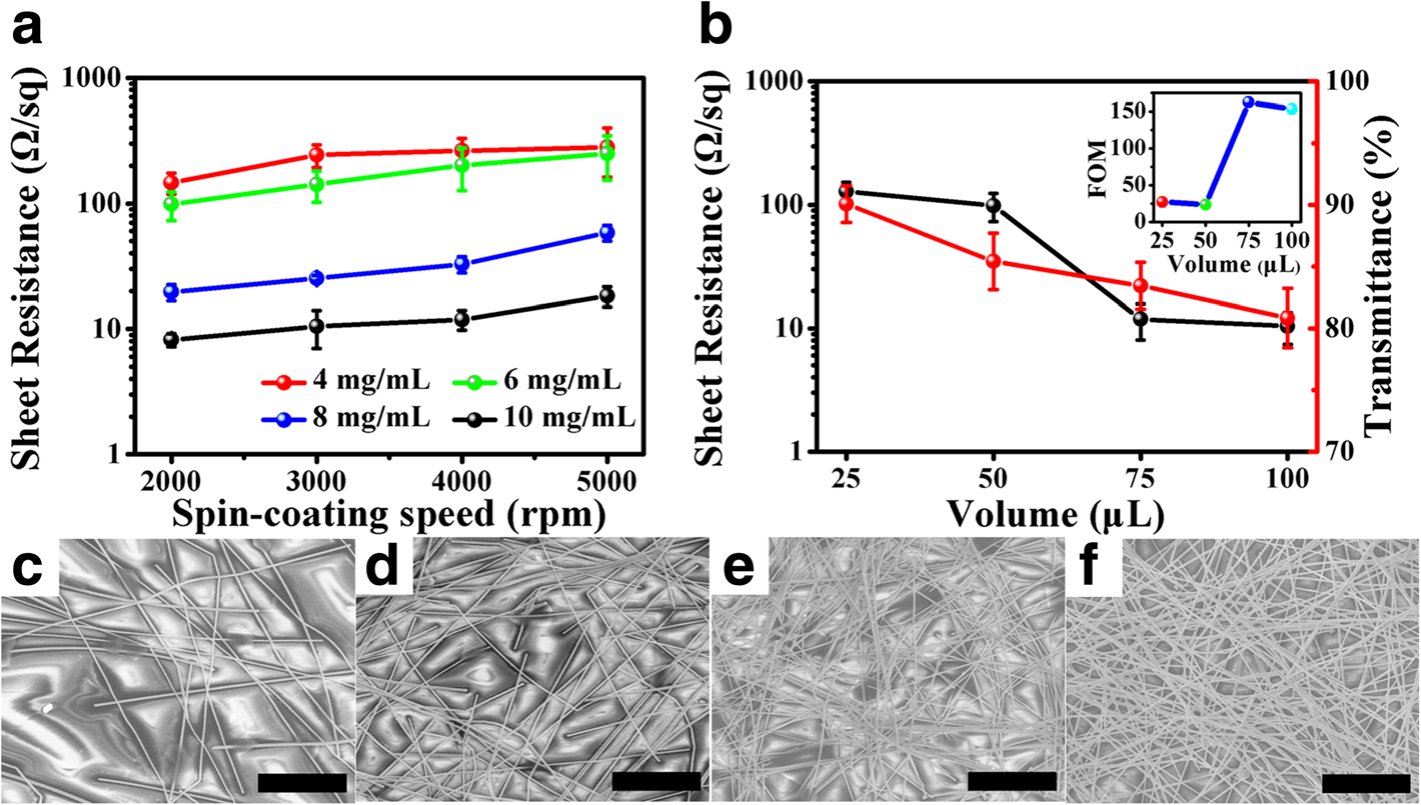
**a** Sheet resistance of Ag NWs films vs the spin-coating speed at different concentration of Ag NWs. **b** Comparison of optoelectronic performance of Ag NTEs fabricated by different volume of Ag NWs solutions. The concentration of Ag NWs solution is 6 mg/mL, and the volume of each spin coating is 25 μL. The *inset* is the FOM values of Ag NWs films vs the volume of Ag NWs solution. **c**–**f** SEM images of Ag NWs films fabricated by different volumes of Ag NWs solutions, **c** 25 μL, **d** 50 μL, **e** 75 μL, **f** 100 μL, respectively. All the *scale bars* are 5 μm

It is necessary to optimize the spin-coating process to fabricate high-quality Ag NWs films. As shown in Fig. 5a, it is observed that the sheet resistance increases as increasing the rotational speed because the number of Ag NWs clinging on the surface of PET decreases, resulting in the decline of conductivity. In addition, it is noteworthy that the sheet resistance significantly decreases to 19.6 Ω/sq when using the 8 mg/mL of Ag NWs solution. And it decreases almost fivefold compared with that of using 6 mg/mL, which could be attributed to the formation of more efficient conductive percolation routes in the Ag NWs network, whereas some macroscopic agglomerates of Ag NWs appears as the concentration increases to 8 mg/mL. Then, the repeated spin-coating process is carried out. As shown in Fig. 5b, both the transmittance and sheet resistance decrease as increasing the times of spin coating. More importantly, when the volume of Ag NWs solution is added from 50 to 75 μL, the sheet resistance dramatically decreases from 98.46 to 11.87 Ω/sq. As the volume further increases to 100 μL, the sheet resistance decreases to 10.42 Ω/sq with a transmittance of 80.95%. It indicates that the density of nanowires in the nanostructured transparent conducting networks may reach the tipping point where the transition from percolation behavior to bulk behavior occurs [81], when the volume is added to 75 μL. Moreover, to evaluate the performance of NTEs, the figure of merit (FOM) is calculated that correlates transmittance with sheet resistance. Generally, the transmittance (*T*_*λ*_) and sheet resistance (*R*_*s*_) of a thin metallic film satisfy the following Eq. (1):

1 $$ {T}_{\lambda }={\left(1+\frac{188.5}{R_{\mathrm{S}}}\frac{\sigma_{\mathrm{op}}\left(\lambda \right)}{\sigma_{DC}}\right)}^{-2} $$

*σ*_op_ *(λ)* is the optical conductivity and *σ*_DC_ is the direct current conductivity of the film [37]. The value of *σ*_DC*/*_ *σ*_op_ *(λ)* are employed as FOM. And a higher value of FOM means better optoelectronic performance. The inset in Fig. 5b exhibits the FOM values of NTEs fabricated by different volume of Ag NWs solutions. When the volume is added to 75 μL, the Ag NWs has the highest FOM value, increasing dramatically from 23.3 to 162.6. It denotes that the balance is achieved between low sheet resistance and high transmittance when implementing three times of spin coating. In addition, Fig. 5c–f shows the SEM images of Ag NWs films on PET with different densities, corresponding to the volume of Ag NWs solutions for 25, 50, 75, and 100 μl, respectively. From the images, it is obvious that the Ag NWs networks become ever denser and the distribution of Ag NWs is more uniform, as increasing the volume of Ag NWs solution. Therefore, the repeated spin-coating process is available to fabricate uniform Ag nanowire films with various transmittance and sheet resistance for different applications.

For application in NTEs, the nanowire junctions have a significant influence on the conductivity of random Ag NWs network [58]. In polyol process, the as-synthesized Ag NWs retain a residual insulated PVP layer, resulting in high resistance at junctions and the deterioration of conductivity. Lee et al. [59] reported that the repeated solvent washing can reduce the PVP layer from ca. 4 nm to 0.5 nm and allows for room-temperature welding of the overlapping Ag NWs. Similarly, we repeated to wash the as-synthesized Ag NWs for three times with ethyl alcohol to remove the PVP layer as much as possible. As the abovementioned results in Fig. 3a, thin PVP layer with a thickness of 2 nm is left. It can not only efficiently reduce the junction resistance but also ensure the good dispersion of Ag NWs in the solvent. On the other hand, for widthless sticks in two dimensions, the critical number density (*N*_*c*_) of sticks to create a percolation network is given by Eq. (2):

2 $$ {N}_c\times {L}^2=5.71 $$

*L* is the length of nanowires [52]. This equation implies that the number density of Ag NWs required for percolation network is inversely proportional to the square of length. Hence, long nanowires tend to build a sparse and effective percolation network with a low number density. It can not only increase the light transmission but also improve the conductivity through building long percolation routes with less nanowire junctions.

**Fig. 6:**
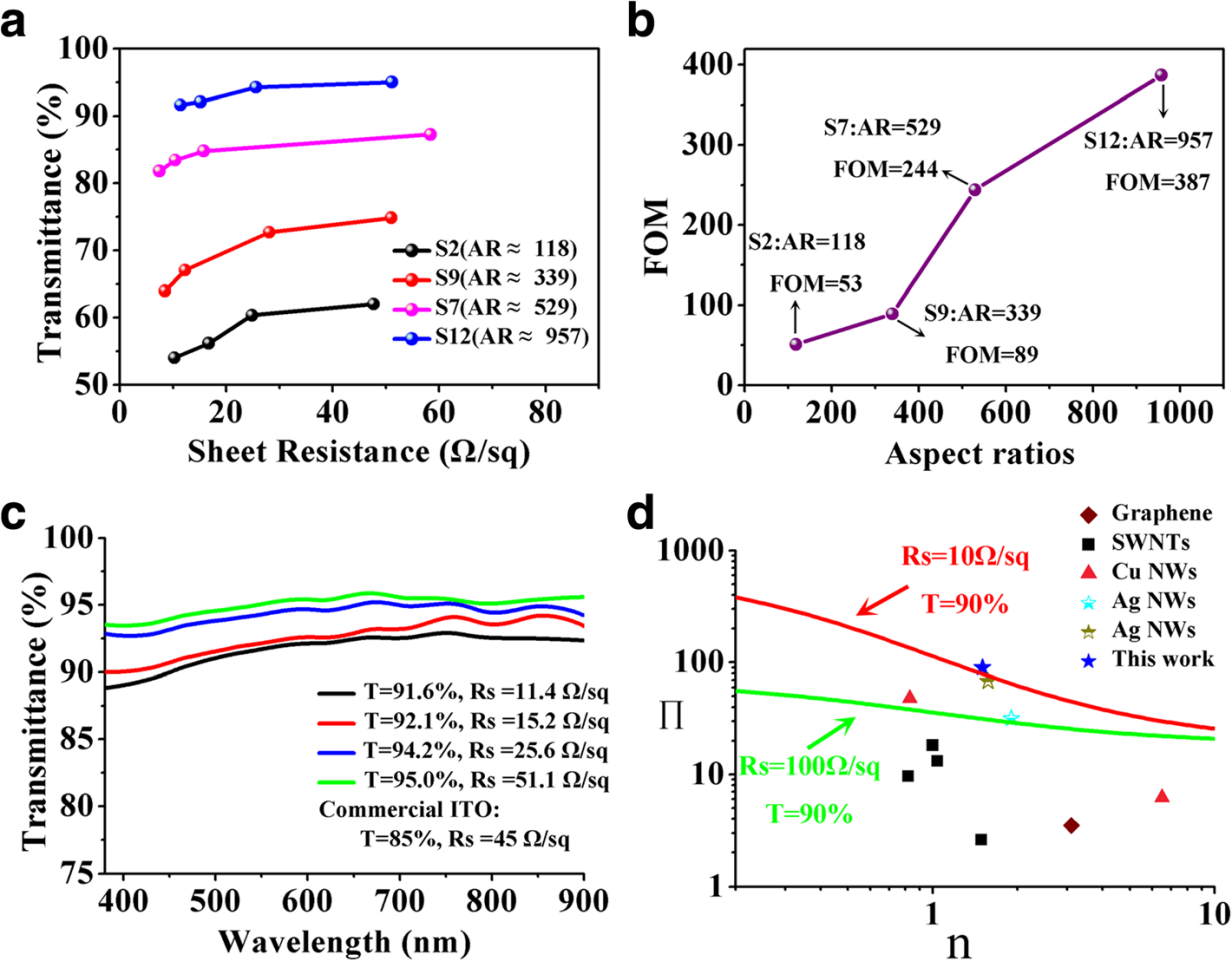
**a** Comparison of optoelectronic performance of NTEs fabricated by Ag NWs with different aspect ratios (AR). **b** The best FOM values of Ag NWs films vs the AR of Ag NWs. **c** The optical transmittance spectra of Ag NWs films fabricated from sample S12. **d** Percolative figure of merit (*П*), plotted against conductivity exponents (*n*). The *solid lines* are plotted at the given combinations of transmittance (*T*) and sheet resistance (*R*_s_), as calculated from eq. (3). The plotted data of graphene, SWNTs, Cu NWs, Ag NWs are from recently published reports [37, 67, 81]. The *star symbol* represents the results of Ag NWs films fabricated using sample S12 from this work

Figure 6a shows the comparison of optoelectronic performance of NTEs fabricated by Ag NWs with different aspect ratios. For samples S2 and S9, the enlargement of parallel transmittance could be attributed to the smaller diameters which reduced from 104.4 to 47.5 nm because nanowires with smaller diameters can scatter less light, leading to a further decrease in haze. As the aspect ratios exceed 500 (sample S7), Ag NWs films with a parallel transmittance of 81.8% (87.2%) and a sheet resistance of 7.4 Ω/sq (58.4 Ω/sq) are obtained. The optoelectronic performance are comparable to those of commercial ITO films (85%, 55 Ω/sq) [5]. Furthermore, when the aspect ratios reach almost 1000 (sample S12), Ag NWs films show superior transmittance (91.6–95.0%) and electronic conductivity (11.4–51.1 Ω/sq) than ITO films. They sufficiently meet the performance requirements of TEs in the application of solar cells or touch screens. Moreover, as shown in Fig. 6b, the biggest FOM value achieves 387, higher than many other reported values of various TEs [62, 73]. The excellent performance could be attributed to the long and thin Ag NWs. In addition, it is noteworthy that the FOM value dramatically increases from 89 to 224 when the aspect ratios enlarge from 339 (sample S9) to 529 (sample S7). The main reason is probably that the longer Ag NWs from sample S7 form a more effective percolation network with a smaller number of nanowires, leading to much more light transmission through the Ag NWs network. It indicates that the long Ag NWs strategy is a facile and effective way to obtain NTEs with promising optoelectronic performance, when the thin Ag NWs with a diameter less than 20 nm are not synthesized successfully [52, 67]. Figure 6c demonstrates optical transmittance spectra of Ag NWs films fabricated from sample S12. The spectra show a wide flat region from visible light to near infrared wavelength, which can improve the utilization range of light and is advantageous for display and solar cell applications, while the transmittance of ITO films displays dramatic fluctuation over the region of visible light [7].

To further evaluate the optoelectronic performance of Ag NWs networks, the percolative FOM, *П*, was proposed in the Eq. (3) by De et al. [81]:

3 $$ T={\left[1+\frac{1}{\varPi }{\left(\frac{Z_0}{R_{\mathrm{S}}}\right)}^{\frac{1}{n+1}}\right]}^{-2} $$

*Z*_*0*_ is the impedance of free space (377 Ω). *T* and *R*_s_ represent the transmittance and sheet resistance of Ag NWs films, respectively. High values of *П* mean low sheet resistance and high transmittance. Percolative FOM (*П*) and conductivity exponent (*n*) in this work are calculated to be 89.8 and 1.50 by using Eq. (3), respectively. The percolative FOM value is higher than other reported values of various TEs (shown in Fig. 6d). It could be attributed to two reasons: The thin PVP layer (ca. 2 nm) can effectively reduce the nanowire junction resistance. On the other hand, the long Ag NWs (ca. 71.0 μm) form long conductive routes in the percolation networks, resulting in the decrease of number of junctions. Interestingly, the value of *n* is a non-universal exponent which has been related to the presence of a distribution of nanowire junction resistance [82–84]. Lee et al. [67] used a laser nano-welding process to reduce the nanowire junction resistance, and the value of *n* is calculated to be 1.57. The value is close to that in our work. It further suggests that the thin PVP layer and long Ag NWs are efficient to allow low-temperature welding of Ag NWs network.

**Fig. 7:**
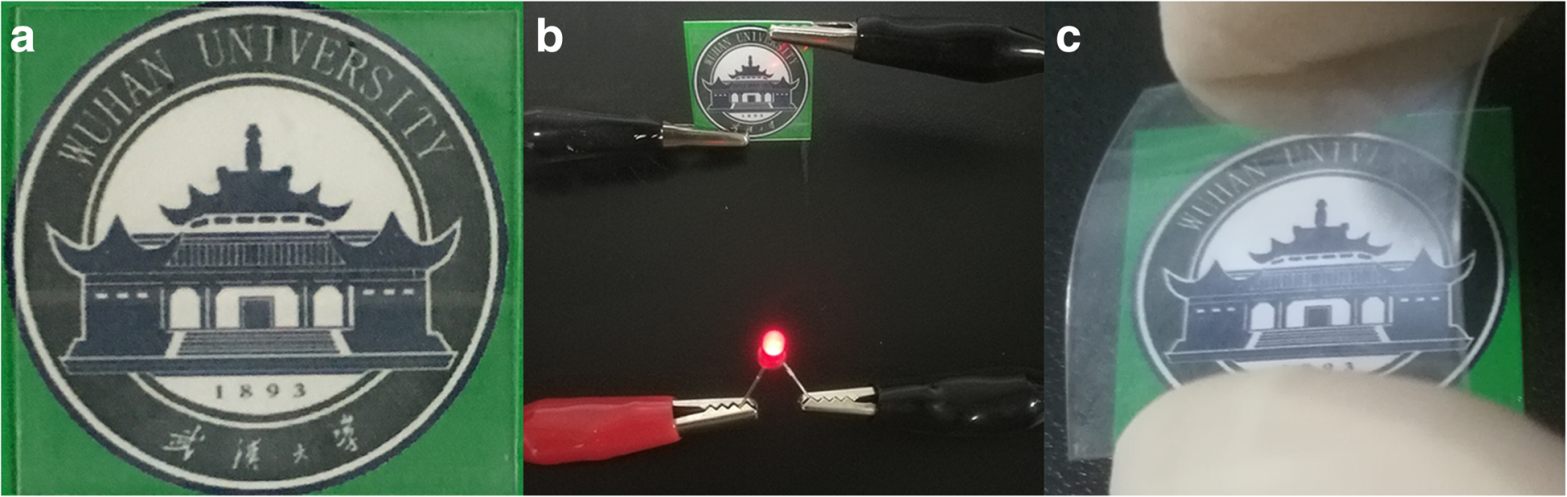
**a** Optical image of as-fabricated Ag NWs films on PET. **b** Ag NWs film is connected in an electric circuit in which an LED is lit. **c** Optical image of the flexible Ag NWs film


Figure 7a exhibits optical photographs of the uniform Ag NWs film on PET. The film is highly transparent as the school badge in the background can be clearly seen through the film. Figure 7b, Additional file 1: Figure S3 and Additional file 2: Video S1 show that Ag NWs film on PET turn on the LED bulb when applying a low voltage. It indicates that the whole surface of Ag NWs film is highly conductive. In addition, The Ag NW film is very flexible as shown in Fig. 7c.

Additional file 2:The video of Ag NWs flexible transparent electrodes. (AVI 9706 kb)


**Fig. 8:**
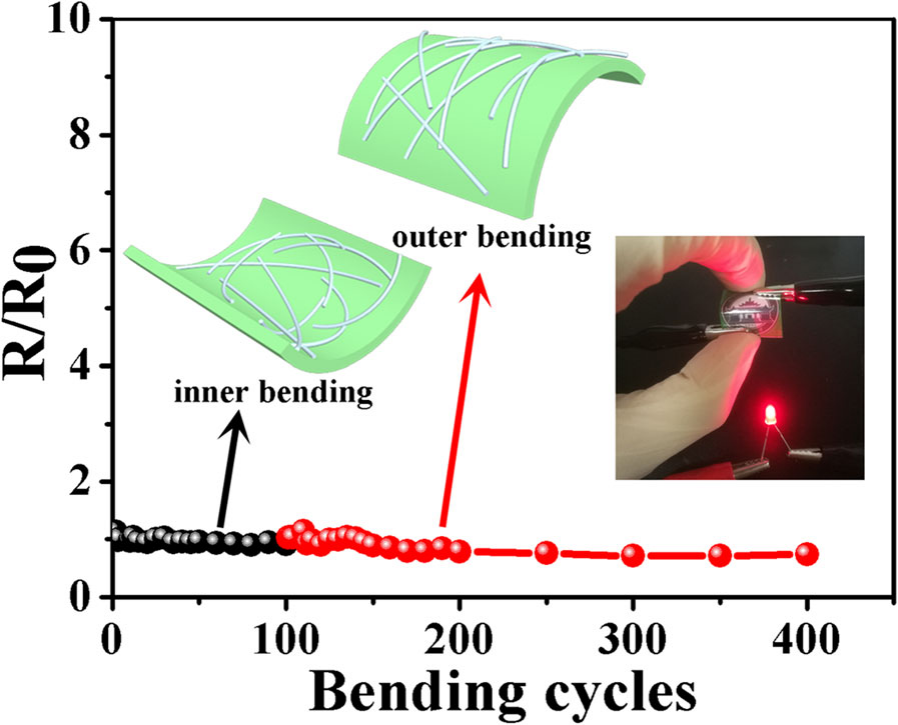
The bending test, including inner bending and outer bending. Both the bending radios are 1.5 cm. The *inset* shows the bent Ag NTEs is still conductive over the whole surface. (*R* and *R*_0_ represent the sheet resistance of films before and after bending test, respectively)

The mechanical stability of the fabricated Ag NTEs on PET substrate is evaluated by a bending test. As shown in Fig. 8, the bending test consists of 100 cycles of inner bending and 300 cycles of outer bending with a bending radio of 1.5 cm. No visible defects, such as cracking or tearing of the surface, are observed even after more than 400 cycles of bending test. And Ag NTEs exhibit a stable electronic performance with little change of sheet resistance. Its property to tolerate hundreds of mechanical bending test could be attributed to the flexibility of long Ag NWs and the benign adhesion to the substrate.

## Conclusions

In summary, Ag NWs with different aspect ratios varying from ca. 30 to ca. 1000 are prepared via a facile PVP-mediated polyol process and are applied to the fabrication of high-performance Ag NTEs with low-temperature sintering. In the polyol process, the diameters of Ag NWs are strikingly reduced and the aspect ratios reach almost 1000 when employing mixed PVP as the capping agent. Additionally, when the aspect ratios exceed 500, the optoelectronic performance of Ag NWs films show good transmittance (81.8–87.2%) and electronic conductivity (7.4–58.4 Ω/sq), comparable to those of commercial ITO films (85%, 45 Ω/sq). Furthermore, high-performance Ag NTEs with a transmittance of 91.6% and a sheet resistance of 11.4 Ω/sq are obtained, as the aspect ratios exceed 1000. The long nanowires and thin PVP layer lead to less number of nanowire junctions and reduced junction resistance, respectively. It allows low-temperature sintering of Ag NWs network, which is advantageous for the applications in the flexible plastic substrates. Moreover, Ag NTEs show excellent flexibility against the bending test. We believe that the ability to synthesize Ag NWs with different aspect ratios and fabricate high-performance NTEs with low-temperature welding are very valuable to the development of flexible electronic devices.

## Additional Files

Additional file 1: Table S1. Reaction parameters of Ag NWs with different concentrations of PVP and mixed PVP molecules at different mole ratios. Herein, silver nanoparticles and silver aggregated nanorods are abbreviated to Ag NPs and Ag ANRs, respectively. **Figure S1.** SEM images of Ag NWs under different reaction conditions: (a) 0.05 M PVP, (b) 0.25 M PVP, (c) 0.55 M PVP, (d) PVP-10, (e) PVP-58, respectively. (f) statistical size distribution of Ag NWs synthesized using PVP-58. **Figure S2.** Statistic sizes distribution of Ag NWs synthesized using different mixed PVP molecules. (a) PVP-40:PVP-58 = 2:1, (b) PVP-40:PVP-58 = 1:1, (c) PVP-40:PVP-58 = 1:2, (d) PVP-40:PVP-360 = 2:1, (e) PVP-40:PVP-360 = 1:1, (f) PVP-40:PVP-360 = 1:2, respectively. **Figure S3.** Ag NWs film is connected in an electric circuit, being applied a low voltage. (DOCX 2653 kb)
