# Supplementary material for: Facile Synthesis of Silver Nanowires with Different Aspect Ratios and Used as High-Performance Flexible Transparent Electrodes
Source: Nanoscale Res Lett. 2017 Aug 7;12:480. doi: 10.1186/s11671-017-2259-6 (PMC5545992; doi:10.1186/s11671-017-2259-6)
Supplement: Supplementary file 2 — Reaction parameters of Ag NWs with different concentrations of PVP and mixed PVP molecules at different mole ratios. Herein, silver nanoparticles and silver aggregated nanorods are abbreviated to Ag NPs and Ag ANRs, respectively. Figure S1. SEM images of Ag NWs under different reaction conditions: (a) 0.05 M PVP, (b) 0.25 M PVP, (c) 0.55 M PVP, (d) PVP-10, (e) PVP-58, respectively. (f) statistical size distribution of Ag NWs synthesized using PVP-58. Figure S2. Statistic sizes distribution of Ag NWs synthesized using different mixed PVP molecules. (a) PVP-40:PVP-58 = 2:1, (b) PVP-40:PVP-58 = 1:1, (c) PVP-40:PVP-58 = 1:2, (d) PVP-40:PVP-360 = 2:1, (e) PVP-40:PVP-360 = 1:1, (f) PVP-40:PVP-360 = 1:2, respectively. Figure S3. Ag NWs film is connected in an electric circuit, being applied a low voltage. (DOCX 2653 kb) [file 11671_2017_2259_MOESM1_ESM.docx]

Supporting Information

Facile synthesis of silver nanowires with different aspect ratios and used as high-performance flexible transparent electrodes

Qingwen Xue^1^, Weijing Yao^1^, Jun Liu^1^, Qingyong Tian^1^, Li Liu^1^, Mengxiao Li^1^, Qiang Lu^1^, Rui Peng^1^ and Wei Wu^1,2^

^1^ Laboratory of Printable Functional Nanomaterials and Printed Electronics, School of Printing and Packaging, Wuhan University, Wuhan 430072, P. R. China

^2^ Shenzhen Research Institute of Wuhan University, Shenzhen 518057, P. R. China

Email address:

Wei Wu, [weiwu@whu.edu.cn](mailto:weiwu@whu.edu.cn) (Corresponding author)

**Table S1** Reaction parameters of Ag NWs with different concentrations of PVP and mixed PVP molecules at different mole ratios. Herein, silver nanoparticles and silver aggregated nanorods are abbreviated to Ag NPs and Ag ANRs, respectively.

| Sample | PVP  /mol | mixed PVP molecules  (molar ratio) | Morphology | Diameter(D)  /nm | Length(L)  /μm | Aspect ratio(L/D) |
| --- | --- | --- | --- | --- | --- | --- |
| S1 | 0.05 | PVP-40 | Ag NPs | / | / | / |
| S2 | 0.15 | PVP-40 | Ag NWs | 104.4±17.3 nm | 12.3±5.2 μm | ~118 |
| S3 | 0.25 | PVP-40 | Ag NWs+ Ag NPs | / | / | / |
| S4 | 0.55 | PVP-40 | Ag NPs |  |  |  |
| S5 | 0.15 | PVP-10 | Ag NPs+ Ag ANRs | / | / | / |
| S6 | 0.15 | PVP-58 | Ag NWs | 235.0±67.3 nm | 6.7±2.8 μm | ~28 |
| S7 | 0.15 | PVP-360 | Ag NWs | 132.1±27.1 nm | 69.9±27.0 μm | ~529 |
| S8 | 0.15 | PVP-40:PVP-58=2:1 | Ag NWs | 137.9±24.1 nm | 18.2±5.5 μm | ~132 |
| S9 | 0.15 | PVP-40:PVP-58=1:1 | Ag NWs | 47.5±7.4 nm | 16.1±5.5 μm | ~339 |
| S10 | 0.15 | PVP-40:PVP-58=1:2 | Ag NWs | 222.8±59.6 nm | 11.5±3.7 μm | ~52 |
| S11 | 0.15 | PVP-40:PVP-360=2:1 | Ag NWs | 74.9±18.2 nm | 48.2±12.6 μm | ~643 |
| S12 | 0.15 | PVP-40:PVP-360=1:1 | Ag NWs | 74.2±12.0 nm | 71.0±16.0 μm | ~957 |
| S13 | 0.15 | PVP-40:PVP-360=1:2 | Ag NWs | 76.6±16.6 nm | 67.9±18.0 μm | ~886 |


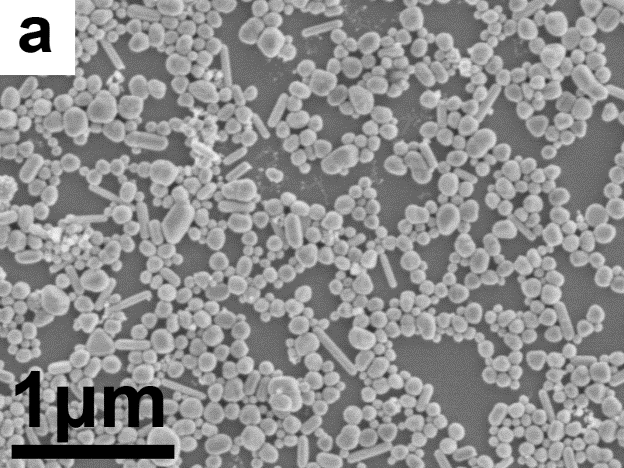

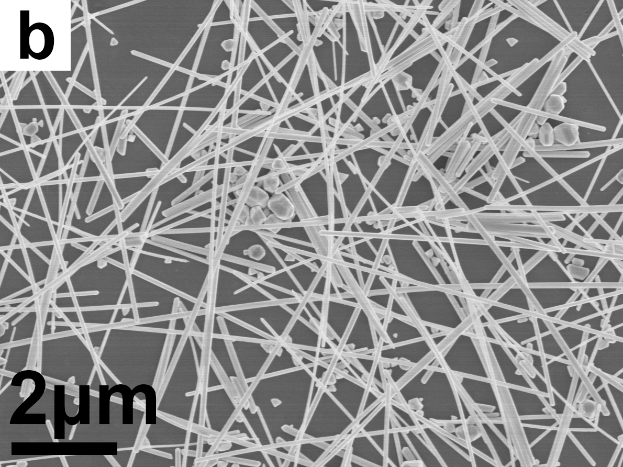


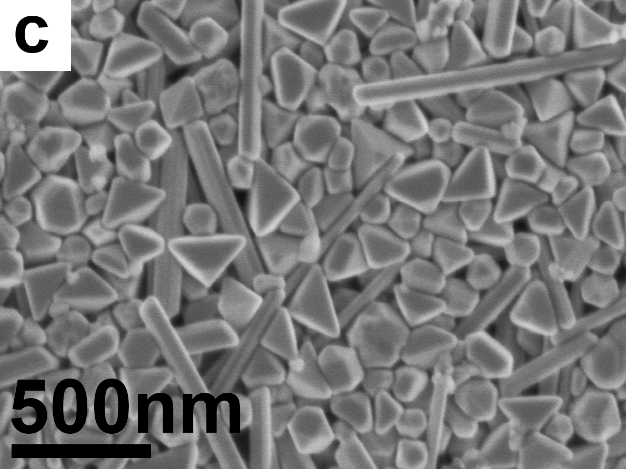

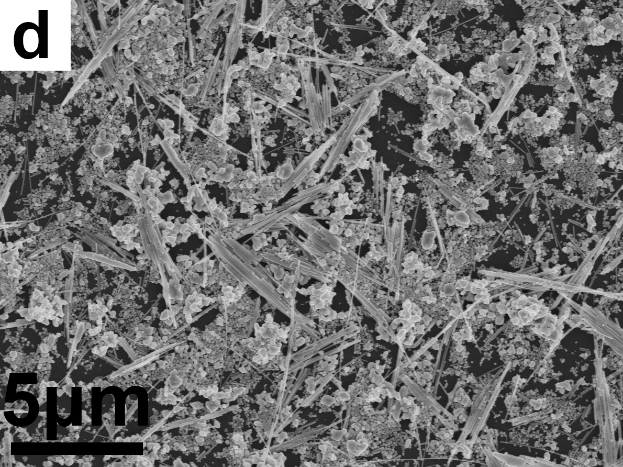

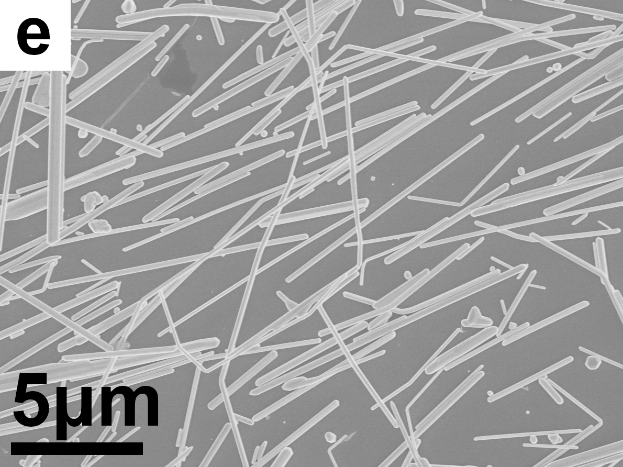

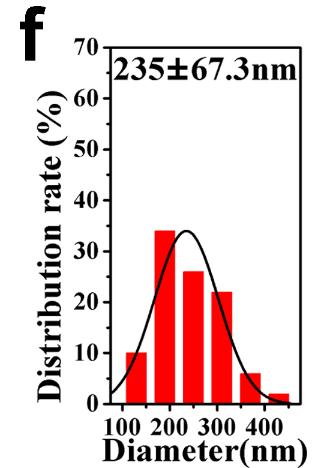

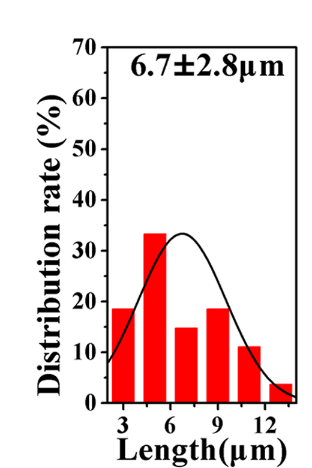


**Figure S1** SEM images of Ag NWs under different reaction conditions: (a) 0.05 M PVP, (b) 0.25 M PVP, (c) 0.55M PVP, (d) PVP-10, (e) PVP-58, respectively. (f) statistical size distribution of Ag NWs synthesized using PVP-58.


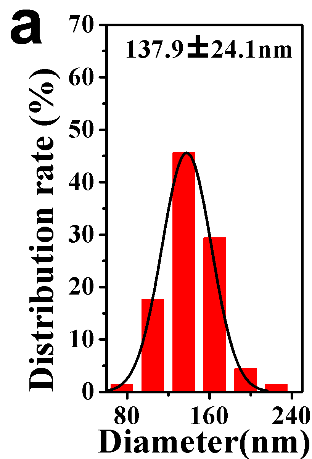

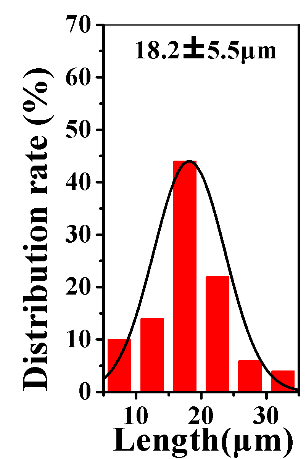

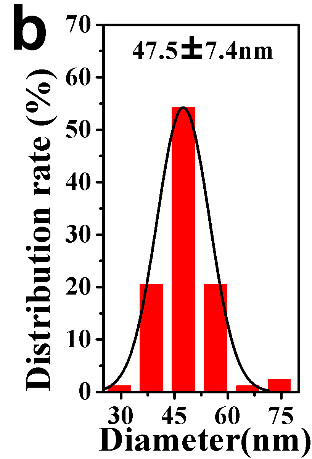

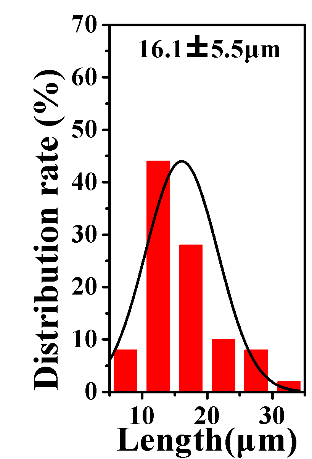


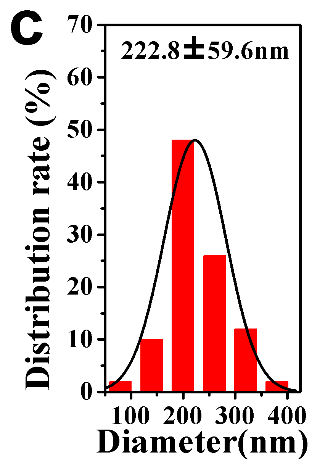

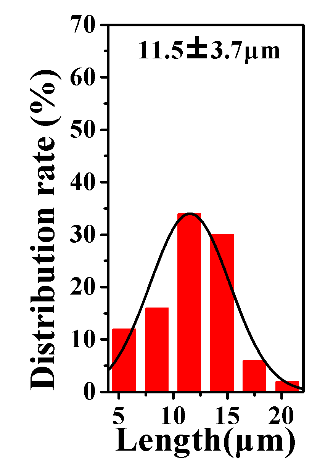

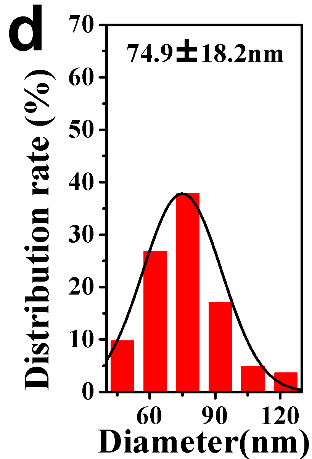

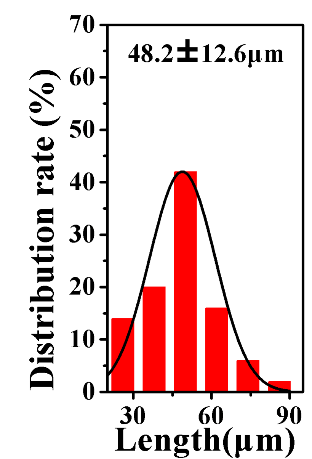


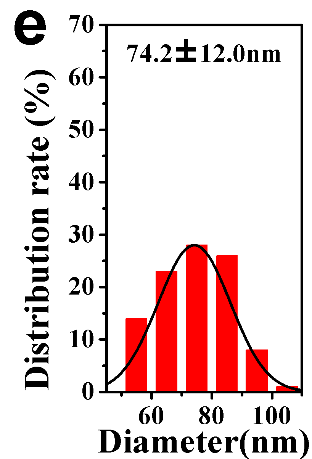

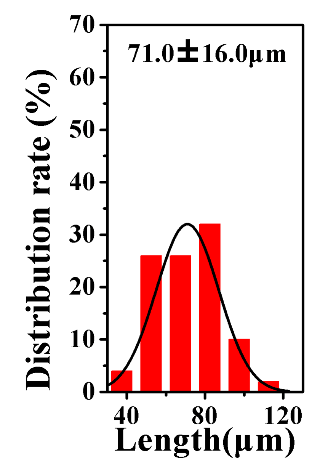
**
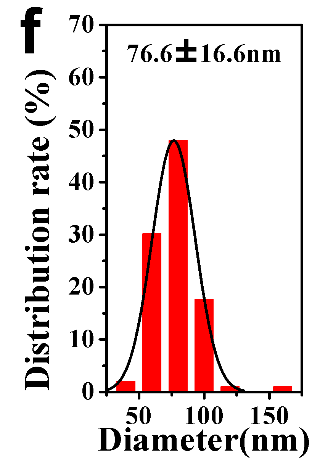
**
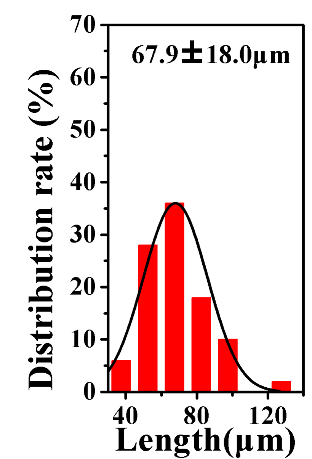


**Figure S2** Statistic sizes distribution of Ag NWs synthesized using different mixed PVP molecules. (a) PVP-40:PVP-58=2:1, (b) PVP-40:PVP-58=1:1, (c) PVP-40:PVP-58=1:2, (d) PVP-40:PVP-360=2:1, (e) PVP-40:PVP-360=1:1, (f) PVP-40:PVP-360=1:2, respectively.


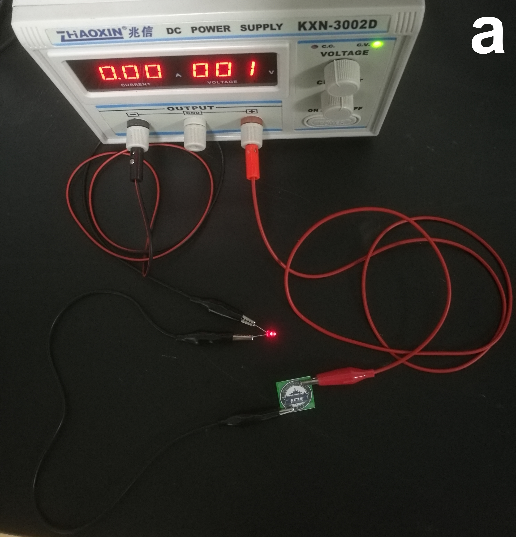

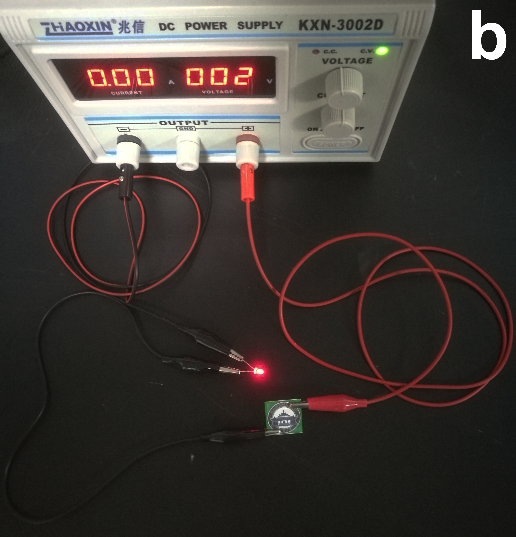


**Figure S3** Ag NWs film is connected in an electric circuit, being applied a low voltage.
